# Doctors’ perceptions of using their digital twins in patient care

**DOI:** 10.1038/s41598-023-48747-5

**Published:** 2023-12-07

**Authors:** Mohan Zalake

**Affiliations:** Biomedical and Health Information Sciences, University of Illinois Chicago, Chicago, 60601 USA

**Keywords:** Patient education, Computer science

## Abstract

Recent Artificial Intelligence (AI) advancements have facilitated tools capable of generating digital twins of real human faces and voices for interactive communication. In this research, we explore utilizing Digital Twins of Doctors (DTDs) in healthcare because using a doctor’s identity can provide benefits like enhancing the credibility of the health information delivered using computers. DTDs are computer-controlled AI-generated digital replicas of doctors that closely resemble their characteristics. However, there exist limitations, including the social implications of using a doctor’s identity, potential negative impacts on doctor–patient communication, and liability concerns. To ensure a comprehensive understanding of DTD usage in healthcare before widespread adoption, systematic research is essential. As a step towards this direction, in this qualitative research, we report findings from 13 semi-structured interviews with doctors. Our findings indicate that doctors believe DTDs offer benefits by saving doctors’ time through the efficient delivery of repetitive information and personalizing patient care. Moreover, while using a doctor’s identity can enhance credibility, it also raises concerns about using a doctor’s identity to spread potential misinformation. These findings contribute by informing future researchers about doctors’ perspectives on utilizing DTDs in healthcare, guiding the development of effective implementation strategies for responsible DTD integration into healthcare.

## Introduction

Recent advances in machine learning have led to the development of algorithms and tools that can generate synthetic human characters^1,2^, commonly called Artificial Intelligence (AI)-generated characters. AI-generated characters closely resemble humans through the realistic rendering of faces^1^, voices^3^, emotions^4^, and behaviors^5^. Among these AI-generated characters, a subset known as ‘deepfakes’ has gained notoriety^6^. Deepfakes are AI-generated characters that depict real humans, often with uncanny accuracy in mimicking their facial features and voices. Deepfakes are created using generative AI, a type of AI that is capable of generating text, images, or other media using generative machine learning models. While the term ‘deepfake’ is often associated with the misuse of this technology for deceptive purposes, it can also be used constructively. One such constructive application in healthcare context is the creation of Digital Twins of Doctors (DTD). DTD is a digital replica of a doctor that resembles a real doctor.

DTDs can be used as embodied conversational agents (i.e., characters controlled by a computer) in healthcare. The benefits of using embodied conversational agents in healthcare are well-documented in the literature^7–9^. Embodied conversational agents allow the simulation of face-to-face interactions which enables the development of trust^10^, rapport^11^, and engagement^12^ with patients and thereby improve patient communication^13^ and satisfaction^14^. However, embodied conversational agents require significant resources and effort in designing^15^. Designers have to make several design decisions such as appearance^16^ and attire^17^ that can affect people’s perceptions of an agent (e.g., credibility and expertise^17^). A potential solution to address the challenges in designing embodied conversational agents is to use DTDs. By using a DTD as an embodied conversational agent that has high realism and shares a resemblance (e.g., facial and voice) with a patient’s own doctor, designers can potentially avoid the challenges in designing agents. More importantly, embodied conversational agents may benefit from sharing a resemblance to the patient’s doctor as patients may associate certain qualities of doctors with qualities of agents (e.g., higher trust or perceived expertise) due to shared appearance.

Beyond benefits to the design of embodied conversational agents, using AI-generated characters as DTDs provides valuable opportunities in several aspects of patient healthcare. For example, DTDs can be used to augment existing practices of patient education wherein DTDs can be used to provide deeper interactions with patients (e.g., interpreting different aspects of lab results) which is often not possible during their short-duration one-to-one sessions with doctors^18^. DTDs can also help prepare patients in discussing stigmatizing topics (e.g., sexually transmitted diseases) before they visit doctors by allowing patients to practice discussing uncomfortable topics or by providing examples of simulated conversations with DTDs. Patients may also feel comfortable during one-to-one discussion sessions with their doctors as they have previously interacted with a familiar face (i.e., DTD). Along with patients, doctors and nurses who are often time-constrained^18^ can also benefit from DTDs as their workload can be reduced by delegating certain tasks to DTDs.

While many potential opportunities exist in using DTDs, understanding their limitations is also important. The use of DTDs can have both social and legal implications. For instance, the synthesized media with DTDs may be tampered with to spread misinformation or promote harmful behaviors^19^, or distort expert opinions which would lead to misportrayal of the doctors. Another potential limitation is that overuse of DTDs may further reduce doctor–patient communication which is already a concern in existing patient care^18^. Further, if DTDs are used as a replacement tool for healthcare professionals rather than as a supplementary tool, then reduced human–human communication may negatively affect patient mental health^20^. In addition, when harm is caused by DTDs, legal concerns about whom to blame (patient or programmer or doctor) may also arise.

Given there exist both potential benefits and limitations to using DTDs in patient care, research efforts are required to systematically study the implications of using DTDs in healthcare before widely adopting them^2^. Prior studies have undertaken investigations to explore doctors’ perceptions regarding the adoption of related technologies, such as chatbots^21,22^ and avatars^23^, shedding light on the potential benefits and specific constraints within the healthcare domain when embracing conversational technologies. However, further research is warranted for DTDs due to the utilization of doctors’ identities in creating their digital autonomous counterparts which may elicit diverse responses, akin to the public perception of deepfakes^24^. Researchers have urged to study the social implications surrounding deepfake technology which involves understanding perceptions of all the relevant stakeholders before adopting them^19^. Therefore, in this work, we take the first step by examining the perceptions of doctors in potentially utilizing DTDs in healthcare. Understanding doctors’ perceptions about DTDs is critical to determining the adoption of DTDs by doctors. The research would also help understand if researchers and doctors have a similar understanding of potential opportunities and concerns regarding DTDs usage. With such an understanding, future researchers will be better positioned in identifying the design and responsible usage of DTDs in patient care. We address the following research question: *What are doctors’ perceptions of using DTDs in patient care?*

## Method

### Research design

The study was approved by the University of Illinois’ institutional review board. All methods were carried out in accordance with relevant guidelines and regulations. In the study, semi-structured interviews were conducted with doctors and medical residents from the University of Illinois Health. Semi-structured interviews were chosen as the data-gathering method because semi-structured interviews can help explore and go deeper into understanding doctors’ perceptions, feelings, and ideas regarding DTDs. In addition, the semi-structured individual interviews would allow participants to have ample time to comprehend the concept of digital twins and interview topics and express their thoughts more effectively. Two pilot sessions were conducted with another researcher and a physician to test if the video used in this study provided sufficient details about digital twins to participants and to revise the study materials.

**Figure 1:**
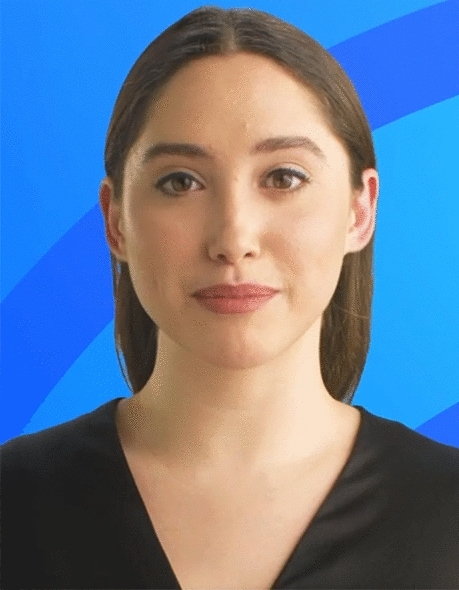
Image from the video of an AI-generated character shown to participants in the study.

The study took place virtually on the Zoom video conferencing platform. Each session was restricted to a 60-minute duration. All the participants were informed that their participation is entirely voluntary, and are free to withdraw from the study at any time for any reason. Participants were informed that identifiable information will not be used for publication. We confirm that informed consent from all subjects was obtained for the publication of anonymized information in a publication. After participants consented to participate in the study, participants were asked questions related to their current practices in discussing health information with patients which involved questions about their background, role, workload, a typical patient session, and the situations when they felt time pressure or had to prioritize information. Due to the novelty of the technology, it was expected that not all participants would be aware of the technology’s capability and verbal explanation might not be sufficient to demonstrate the technology’s capability. Therefore, a video was shown to doctors to provide details on what digital twins are and how they are created. A short 1–2 minute video demonstrated a human-like virtual character (see Fig. 1) discussing a made-up lab test result in English. The participants were also shown how the video was created using the commercial platform Synthesia^25^. This helped participants not only understand the new technology but also visually see what digital twins were and helped us gather more naturalistic perceptions of the participants grounded in a real-world tangible example. After the video was shown, the participants were asked to imagine if a similar video was created using their identity. Participants were then asked about their familiarity, thoughts, concerns, and potential use cases of digital twins. The interview also included questions on how the doctor would introduce and explain digital twins to their patients, how they would handle any patient feedback or complaints, and if they would be interested in creating their own digital twin for patient communication. To mitigate interviewer bias, an interview guide was developed and used. The interview guide helped to keep questions consistent across interviewees. Follow-up questions were included in the guide to help the interviewer stay on track and bring back the focus on the study. The questions were framed to be open-ended and not leading questions. The interview guide and video are attached with the Supplementary materials.

### Participants

To be eligible to participate in the study, the participant was required to be a physician or a medical resident who interacted with patients at least once a week. Participants were recruited via emails to hospital and university listservs. In total, 13 participants (7 male and 6 female) participated in the study. There were 10 attending physicians, 2 residents, and 1 fellow with a range of medical specialities, including family medicine, internal medicine, pediatrics, bariatrics, radiology, immunology, neurosurgery, plastic surgery, critical care medicine, orthopedics, and urology. Doctors’ medical experience ranged between 1 and 35 years with a mean of 19.7 years. The distribution of participants across age groups was as follows: 25–34 (n = 3), 35–44 (n = 1), 45–54 (n = 5), 55–64 (n = 3), and 65 + (n = 1). The sample size was determined based on the data saturation in the collected data^26^. Data collection was completed once no new information was obtained from additional interview sessions.

### Data analysis

For the analysis, all the interviews were transcribed, and participant identities were anonymized. The interview transcripts were analyzed using reflexive thematic analysis^27^ using QualCoder^28^. The reflexive thematic analysis provides a method to develop, analyze and interpret patterns across a qualitative dataset while acknowledging the researcher’s subjective perspectives. Using the researcher’s previous experience and knowledge for a comprehensive understanding of the qualitative data is an important component in reflexive thematic analysis. To incorporate reflexive thematic analysis in our research, the lead researcher’s expertise in utilizing embodied conversational agents in healthcare was used to develop themes that are relevant in investigating the use of DTDs as embodied conversational agents in healthcare. The process involved identifying concepts by labeling content from transcripts and then defining and developing themes based on their properties. Only the lead researcher coded all the transcripts as it is acceptable to have a single coder for reflexive thematic analysis^29,30^.

## Results

Our main goal was to explore the doctor’s perceptions of using DTDs in healthcare. Therefore, in this paper, we only discuss identified themes and codes associated with the research question described in this paper. The themes not relevant to the research question will be explored and discussed in our future work. The codes (n = 52), sub-themes (n = 5), and key themes (n = 5) relevant to this research were derived from all the interview transcripts (n = 13) and are described in Tables 1, 2, 3, 4, and 5. The code frequencies in each table were calculated based on the number of times a code has appeared across all interviews, allowing us to see the prevalence of a code across the entire interview data. A single interview may be counted multiple times depending on the number of times code has appeared in the interview. Along with the code frequency, we have also noted how many distinct participants talked about the code when discussing them in the following sections. The percentages in each table represent the proportion of each code’s occurrence relative to the total number of occurrences of all codes in a given theme.

### Theme 1: Benefits of using DTDs in patient care

One prominent theme that emerged was doctors’ perceptions of the potential benefits of DTDs in patient care. This theme encompassed a total of 19 distinct codes (see Table 1), each representing a specific perceived advantage associated with DTD utilization. We discuss some of the frequently discussed benefits below.

**Table 1:** Codes in Theme 1 (benefits) with their description and frequency.

Codes	Description	Code frequency (% occurence in theme)
Accessibility	Benefits related to the accessibility of health information outside the medical setting	3 (2.83%)
Charting	Benefits related to collecting patient health information	3 (2.83%)
Describe other complications	Benefits related to discussing complications of a medication or a procedure	6 (5.66%)
Disease information	Benefits related to providing information about the disease for which patient is being treated for	7 (6.6%)
Follow Up with patients after visit	Benefits related to following up with patients after doctor visits for next steps	6 (5.66%)
Group information sessions	Benefits related to providing group sessions for patients	1 (0.94%)
Improve the credibility of information	Benefits related to improving the credibility of information presented to patients	5 (4.72%)
Patient check in/check out	Benefits related to performing patient check-in and check-out	2 (1.89%)
Patient medical history	Benefits related to collecting patient medical history	2 (1.89%)
Patient’s own time and space	Benefits related to patients accessing health information based on their convenience	3 (2.83%)
Personalized communication	Benefits related to personalizing communication with patients	14 (13.21%)
Post-operation	Benefits related to providing post-operation instructions	4 (3.77%)
Pre-operation	Benefits related to providing pre-operation instructions	10 (9.43%)
Prepare for doctor’s visit	Benefits related to preparing patients for doctor’s visit	4 (3.77%)
Preventive care messages and appointments	Benefits related to providing preventive care information to patients	2 (1.89%)
Reminders	Benefits related to sending reminders to patients	3 (2.83%)
Repetitive information	Benefits related to providing information that is repetitive across multiple patients	12 (11.32%)
Test instructions, interpretation, and education	Benefits related to providing medical test instructions, interpretation of test results, and educating patients to address concerns from test results	16 (15.09%)
Visual demonstration	Benefits related to providing visual demonstration to patients	3 (2.83%)

Doctors (n = 9) found DTDs could be beneficial in efficiently delivering repetitive information that doctors have to deliver between multiple patients. One doctor emphasized the repetitive nature of delivering instructions, stating—“*Instructions like these I spend a lot of time saying the same thing again and again... to every single patient that I see. That sometimes I feel like I’ve become a robot now. So I think, having this AI deliver all the instructions in a ready, detailed manner. ... I think, would help us*” [P0957]. Doctors suggested DTDs can perform repetitive discussions like instructions, interpretation, and education of medical tests (n = 9) or pre/post-operative tests (n = 7). For example, a doctor suggested—“*giving them pre-op instructions like before you come for the procedure, come on with an empty stomach, make sure you don’t, eat or drink anything. Things like that maybe would be helpful to get it delivered this way*” [P0957].

Doctors also suggested using DTDs to share additional information with patients which is usually not possible to discuss in a limited time during patient visits. For example, doctors (n = 5) suggested DTDs can provide additional information to patients regarding the disease, like physiological processes associated with disease or injury. As one doctor described—“*patient could type in and say, what are the long-term effects of lyme disease? How do I know if I’m having those long-term effects, how do I prevent it from happening?*” [P3641]. In addition to providing disease information, doctors (n = 6) suggested using DTDs to share information about potential rare complications associated with a medical procedure or medication with patients. One doctor suggested DTDs can describe complications associated with a procedure in the following way—“*However, there are some complications, bleeding and other things which you may expect.*” [P2628].

In addition to efficiently delivering repetitive and additional information, doctors also suggested opportunities to improve patient care using digital twins. Doctors (n = 7) proposed using digital twins as a personalized approach to communication with patients. One doctor highlighted the potential of sending a personalized video message to review test results, stating—“*Sending a video saying, I reviewed your the results, and everything looks fine. I think people might get more... specialized attention. They might feel more like their doctors... caring for them more because they were able to make this... digital video instead of just sending an email saying, everything looks good... like the doctors [are] spending more time with them*” [P2628]. Doctors also suggested DTDs can enable patients to access health information at their own time and pace (n = 3) while improving the accessibility of information to people in regions who have difficulty accessing healthcare and have language barriers (n = 2). As one doctor suggested—“[DTDs] *may help people that are in rural areas or have difficulty accessing clinic*” [P8363].

Other suggestions were related to DTDs performing some of the tasks performed by either doctors or medical staff such as filling out medical forms (n = 3), collecting patient history (n = 2) following up with patients after the visit (n = 5), patient check-in and check-out procedures (n = 2), discussing preventive care services (n = 1), and sending reminders to patients for upcoming appointments (n = 2).

### Theme 2: Concerns about using DTDs in patient care

Along with benefits, doctors also expressed concerns related to the use of DTDs in patient care. This theme encompassed a total of 22 codes (see Table 2). Some of the codes were grouped under sub-themes like legal concerns and technology-related concerns. We discuss some of the frequently mentioned concerns below.

**Table 2:** Codes in Theme 2 (concerns) with their description and frequency.

Sub-themes/codes	Description	Code frequency (% occurence in theme)
Content concerns/accuracy of information	Concerns related to accuracy of the information delivered	17 (11.04%)
Content concerns/content source	Concerns related to source used for developing content	9 (5.84%)
Content concerns/scripted content	Concerns related to scripting the content delivered by the digital twin	5 (3.25%)
Identity-related concerns/use of another face	Doctors describing their perception of using another face rather than using their own face for delivering information to patients	15 (9.74%)
Identity-related concerns/using doctor’s facial identity	Concerns related to using doctor’s facial identity to deliver information to patients	17 (11.04%)
Legal concerns/reasons for expecting legal concerns	Participants providing reasons on why they expect legal concerns	11 (7.14%)
Legal concerns/reasons for not expecting legal concerns	Participants providing reasons on why they do not expect legal concerns	8 (5.19%)
Legal concerns/why doctor’s responsible	Participants explaining why they find doctors responsible if legal concerns arise	6 (3.9%)
Technology-related concerns/appearance	Concerns related to appearance of the digital twin	7 (4.55%)
Technology-related concerns/behavior	Concerns related to behavior of the digital twin	14 (9.09%)
Technology-related concerns/HIPAA	Concerns related to privacy and confidentiality of the patient’s health information	3 (1.95%)
Amount of information to be given	Concerns related to amount of information to be included in the conversation with digital twin	1 (0.65%)
Delivering sensitive information	Concerns related to delivering sensitive information using digital twins	7 (4.55%)
Misinformation	Concerns related to digital twin spreading misinformation	4 (2.6%)
Not beneficial for speciality care	Concerns related to lack of benefits of digital twins in speciality care	5 (3.25%)
Potential repetition/gap between twin and doctor	Concerns related to potential repetition or confusion between digital twin and doctor when doctor doesn’t know what information has been discussed/not discussed by the digital twin	4 (2.6%)
Use for medical advise	Concerns related to process of making a medical recommendation during interaction with digital twin	9 (5.84%)
Use for medical emergencies	Concerns related to using digital twins during medical emergencies	1 (0.65%)
Use more of doctor’s time	Concerns related to using doctor’s time to create content for digital twins	9 (5.84%)
Verbal comprehensibility	Concerns related to comprehensibility of content delivered by the digital twin	2 (1.3%)

A common concern among doctors was the use of a doctor’s facial identity as DTDs would at least share facial identity with doctors. Although a few doctors (n = 4) suggested that using a doctor’s facial identity may improve the credibility of the information presented to patients, several doctors (n = 8) expressed concerns about using their facial identity to deliver misinformation. The contrast is evident in the following quotes from two doctors—“*I think that patients may feel reassured that if they see me ... giving them advice that this is more legitimate*” [P2628] and “*you don’t know if there’s [going to] be wrong communications, wrong information... given to the patient, and if my face will be the one communicating those wrong information, I feel I will be responsible for that*” [P8363]. These quotes illustrate that some doctors may value the trust and rapport that their identity can establish with patients, while others may worry about the ethical and legal implications of being associated with inaccurate or harmful information. Due to concerns associated with using a doctor’s identity, doctors (n = 7) proposed using another or random face to deliver the same information. As one participant described—“*I will feel more comfortable [if] it was just a just randomly generated face that will be talking about labs to the patient. That would be better for me compared to having... my own face there. And the patients, I’m not sure even they would recognize that’s a deep fake.*” [P8363]. This quote shows that some doctors may prefer to use a generic or anonymous face for DTDs, as they may feel less responsible or liable for the information delivered by the agent.

Doctors also expressed several technology-related concerns pertaining to the behaviors and appearance of DTDs and the content delivered by DTDs. A major concern by doctors (n = 9) related to content included the accuracy of the information delivered by DTDs when the content is autonomously generated by computational systems. As one doctor expressed—“*how do you ensure that the information that this digital twin is giving is .... The standard of practice.*” [P2628]. This quote highlights the concern about the reliability and validity of the information provided by DTDs, especially when it is generated autonomously without human oversight. It underscores the need for rigorous quality control mechanisms to ensure that the information aligns with current medical standards and practices. A potential solution to address concerns with autonomously generating content by computational systems is using pre-scripted content. However, doctors (n = 4) anticipated developing scripted content can be a challenging task as it involves anticipating all the patient questions and doctors may have to spend additional time to develop the content (n = 8). This is evident in the following quote—“*just a number of different possible scenarios, is not something that I’m ready to spend time on.*” [P7157]. The quote reflects that while pre-scripted content may address some concerns about accuracy, it may introduce new challenges related to workload and feasibility. In addition, as different sources of information may be used to generate content for DTDs, doctors (n = 4) also voiced their concerns about using their digital twin to deliver information from sources that they disagreed with. For example, one doctor explained—“*How much of an expert is the person who creates this content and ... how close ... he or she is to my practice in terms of mentality, geography, the age, all those things, because even the same topic, people with same degree of knowledge will be presented differently.*” [P7157]. Other than content, doctors expressed concerns about the animation of non-verbal behaviors (n = 6) and the appearance of DTDs (n = 4). Regarding appearance, a doctor said—“*I’m also just thinking about weird, stupid stuff like... I used to always have my beard, and now I just shaved it off, and ... what version of me would patients actually recognize*” [P3087] and regarding behavior, another doctor said—“*I think the facial expressions a little frozen. It’s not as animated as a real human*” [P9832]. This quote suggests that maintaining consistency between a doctor’s real-life appearance and their digital twin’s appearance and behavior could be important for patient acceptance and engagement.

When asked about doctors’ perceptions of potential legal concerns of using DTDs, doctors had mixed perceptions. Doctors (n = 8) expected legal concerns when content from DTD may have led to a loss for patients. As one doctor described—“*That’s the biggest part, and that’s where I think having control is sort of important, like everything that the digital twin says has to be something that’s validated by me. Legal implications are the highest ones of anything.*” [P9212], Whereas some doctors (n = 6) expected no legal concerns as long as relevant disclaimers are provided to patients, content is vetted, and no medical decisions are made by DTD, as one doctor explained—“*I don’t think so. As long as the information is purely educational, it’s not opinion giving*” [P2740]. When asked about who should be responsible if any legal issues arise, several doctors (n = 6) agreed that doctors should be responsible which is evident in the following quote—“*I think ultimately physicians should be responsible at the end of the day. You’ve chosen to use this technology ... to supplement your practice.... ultimately, I think that physicians are responsible... You’ve been given this role of responsibility [in] society for your profession... It’s ultimately why you’re responsible for all the other, like malpractice and legal suits. This is what happens at the hospital*” [P5053].

Doctors also emphasized several instances in which the utilization of DTDs is not advisable. Doctors (n = 5) recommended that emotionally sensitive information should not be conveyed through DTDs, as human empathy and sensitivity is needed in delivering bad news or discussing serious health conditions, as elucidated by a doctor— “*if it’s more sensitive information, as I’m mentioning to you while your cancer has gotten worse. I don’t think that that’s appropriate to use*” [P2628]. Furthermore, doctors specializing in specific areas of medical care (n = 3) emphasized that a substantial portion of the health information conveyed within specialized care is tailored to individual patients. Thus, the utilization of DTDs in specialty care is limited due to the absence of advantages in using DTDs for repetitive information delivery, as described in Theme 1. As a neurosurgeon described—“*it’s just not this same degree of uniformity, as you see you see in patients with a family practice, or like in the chronology, or something like which is very standardized and very straightforward in terms of algorithms. Neurosurgery is more tricky in terms of ... individual treatment choices and the sequence of how they are being developed, and why one thing is not substitute with the other*” [P7157]. To ensure the accuracy and appropriateness of information for each patient, doctors (n = 4) also advised against using DTDs to provide medical advice that is not vetted by a doctor and recommended being transparent about the decision-making process when advice is provided to a patient, as evident in the quote—“*Solutions or offer medical advice ... [like] I would recommend you do this, or try that. I would want to verify that, because you should know what’s already been said to patients when you walk in the door, and because it affects their expectations of the care they receive*” [P5053]. The quote also highlights doctors may value being informed about their digital twin’s interactions with patients, as it could affect their subsequent interactions and relationship with patients.

### Theme 3: Doctors’ perspectives on patient reception

When asked about how patients might perceive DTDs, most doctors (n = 10) predicted positive patient perception. As one doctor suggested patients might appreciate the convenience and accessibility of DTDs, especially given the doctor’s hectic schedules—“*I know people feel that ... doctors are very busy, so they may feel comfortable that this information they’re getting from the digital twin has been approved by their doctor... so I think that they would feel comfortable with it.*” [P2628]. Doctors also expressed some concerns from the perspective of patients. Doctors (n = 9) expect patients may voice concerns on feeling neglected or devalued if their interactions with their doctors are replaced by interactions with DTDs , as one doctor described—“*patients will be like, why can’t you just do it ..., because I’m not valuable enough time wise for you to spend time here, you’re going to go to another patient*” [P2740]. Doctors also suggested lack of patient understanding of the technology may influence patient expectations from DTD. As one doctor described—“*I think, until it becomes... more mainstream. I think there may be some confusion as to who they’re interacting with*” [P5990]. Some doctors (n = 7) expect patients may not like DTDs due to lack of understanding of the technology or due to reduced interaction with doctors. As one doctor described a potentially negative scenario—“*... [I] can see that some people will not like it. They will just literally hate it because they will feel like it’s just a substitute for their doctor, and therefore they coming to see doctor end up seeing the robotic assistant and they may not be happy for that.*” [P7157]. Using DTDs can also introduce confusion between doctors and patients as doctors may not be aware of what has already been discussed by DTDs. Doctors (n = 2) expected that this confusion may lead to repetition or omission of information during doctor–patient visits, as described by a doctor—“*I think if the goal is for them to have an opportunity to talk to an AI-generated physician that looks like me, who is supposed to be my digital twin. Then does that mean that they will no longer feel like they have to ask me the questions, or are we just going to repeat everything? In which case it offers no additional functionality.*” [P5053]. Some doctors (n = 3) also expect that there may be negative reception during early adoption but expect that patients will adapt as technology becomes more ubiquitous. As one doctor described—“*I think over time as the technology becomes more facile and becomes more present in people’s lives, they’ll accept it more. Right now, ... I don’t talk to a digital assistant at the bank. I don’t have a digital twin of my bank... I don’t think anyone’s really used to this technology. And so at the beginning, it’s going to be creepy.*” [P9832].

**Table 3:** Codes in Theme 3 (patient perceptions) with their description and frequency.

Codes	Description	Code frequency (% occurrence in theme)
Positive patient reception	Doctors expecting that patients will positively perceive digital twins	15 (28.85%)
Negative patient reception	Doctors expecting that patients will negatively perceive digital twins	7 (13.46%)
Patient have to adapt	Doctors expecting patient would have to adapt to interaction with digital twins	4 (7.69%)
Patient understanding of the technology	Concerns related to patient’s understanding of digital twin technology	11 (21.15%)
Preference for human interaction	Concerns related to patient preference for human interaction rather than with digital twin	15 (28.85%)

### Theme 4: Financial considerations

Doctors proposed different financial models for costs associated with developing and deploying digital twins. Doctors (n = 2) suggested billing for the doctor’s time in developing and vetting the content, as one doctor described—“*returning a message to a patient,.. If it takes a certain amount of time you can bill for it. So I think that this should be similar to that*” [P0847]. Whereas another doctor suggested billing patients to talk to doctors can motivate patients to use DTDs that can be freely made available by hospitals, as described—“*I think at some point in the future doctors time will be compensated for all these activities, so the patient might have to pay more to ... have a discussion with their doctor. ... I think at some point we’re [going to] have to start charging patients for clinician time. ... that’ll make patients more accepting of other formats of education. They’ll have a financial incentive not to have to talk to their doctor every time.*” [P7813].

**Table 4:** Codes in Theme 4 (financial considerations) with their description and frequency.

Codes	Description	Code frequency (% occurance in theme)
Bill for doctor’s timedeveloping content	Doctors suggesting billing for time spent in developing digital twin content	3 (42.86%)
Bill to talk to doctor	Doctors suggesting to bill patients to talk to doctor as an alternate to talking to digital twins at no cost	3 (42.86%)
Hospital pays for costs	Doctors suggesting hospitals to cover the costs of developing and deploying digital twins	1 (14.29%)

### Theme 5: Willingness to use

When asked about doctors’ willingness to create their own DTDs and use them in their own practice to communicate with patients, most doctors (n = 11) agreed, if certain criteria were met. Criteria involved patient reception of the technology, efforts associated with the DTD development, and how it affects healthcare outcomes. As one doctor described the criteria—“*How easy is it to get good content to generate it. I want to ... definitely need to know the patient’s satisfaction with it. What their thoughts are. I would love eventually to get the harder outcomes of, does it actually increase the rate of preventative services? Does it .. do what we’re hoping it? Its intended purpose is to drive a higher rate of high-quality care or what’s considered the gold standard, or the standard of care or evidence-based guidelines or quality measures.*” [P3087]. Doctors cited potential benefits to patients (n = 5) and their openness to adopt new technologies (n = 4) as motivations for embracing DTDs.

**Table 5:** Codes in Theme 5 (willingness to use) with their description and frequency.

Sub-themes/codes	Description	Code frequency (% occurance in theme)
Why yes/adopt new technologies	Doctors open to use digital twins in their own practice because they want to adopt new technologies in their practice	4 (11.43%)
Why yes/benefits patients	Doctors open to use digital twins in their own practice because it would be beneficial to patients	5 (14.29%)
Why yes/saves time	Doctors open to use digital twins in their own practice because it would save them time	1 (2.86%)
Acceptance criteria	Doctors describing criteria that digital twins should meet for them to use digital twins in their own practice	14 (40%)
Yes to willingness to use	Doctors showing willingness to use digital twins in their own practice	11 (31.43%)

## Discussion

The present study is the first to explore doctors’ perceptions of using DTDs in healthcare. The findings from this study expand the prior work on exploring doctors’ perceptions of conversational technologies, such as chatbots and conversational agents^21,22^, by focusing on the unique aspect of using doctors’ identities for DTDs. Our findings revealed doctors’ perceptions on utilizing their identity to create their digital twins, benefits and concerns of implementing DTDs in patient care, patient reception, financial considerations, and their willingness to use DTDs in their own practice. We discuss how these findings contribute to the existing literature on conversational healthcare agents and the implications of using DTDs in healthcare.

The findings from this study contribute to the knowledge of the various opportunities and challenges associated with using DTDs in patient care, thereby broadening the scope of existing literature on using AI in healthcare. As evident in our findings, the benefits included a potential increase in the credibility of the information shared with patients and the opportunities for personalized care as patients can receive information from a familiar face of their doctor. Whereas concerns included the potential to use doctors’ twins to share misinformation and potential disagreement with information sources used to develop content for DTDs. In response to these issues, doctors suggested using a random or other person’s identity instead of a doctor’s identity. This proposition is substantiated by the fact that an AI-based character, without a specific doctor’s identity, can carry out all the advantageous applications proposed by doctors. For example, prior work has used embodied conversational agents to perform similar tasks in the healthcare domain like explaining health documents^31^ or discussing preventive care procedures^32^. Therefore, it is important to evaluate to what extent a doctor’s identity positively contributes to the patient’s conversation with a virtual character. In addition, potential negative consequences may arise from the possibility of errors in interactions with DTDs that could adversely impact doctor–patient relationships, primarily due to the shared identity. Such an understanding can help us evaluate whether additional resources invested in developing DTDs are worthwhile. Researchers would also have to consider what constitutes a digital twin of the doctor beyond physical characteristics (e.g., face and voice). The following questions need further consideration: Should we design DTDs such that their non-physical characteristics like the personality traits represent the personality of a real doctor or an “*ideal*” doctor? How does that influence patients’ perception of their real doctors?

Doctors suggested that DTDs can be utilized to save time by delivering repetitive information to multiple patients. Such information may include standardized medical instructions, pre-and post-operative procedures, and the interpretation of test results, which can be modified as needed for individual patients. However, implementing these in the real world requires addressing several concerns highlighted by doctors like potentially causing confusion between doctors and patients on what has already been discussed by DTD or the amount of time that doctors would need to spend in developing or verifying the content. Similar challenges are faced when developing and verifying content for autonomous agents in the healthcare domain^33^. Current practices to address these issues involve the utilization of credible health information sources and involving healthcare experts^32,34^. However, these practices may not be sufficient in the case of DTDs because one of the causes for doctors’ concerns was due to shared identity with DTD. Due to shared identity, doctors expected information provided by DTDs to align with what doctors would have proposed. This need for alignment can be particularly challenging when doctors disagree with content developed by certain experts or from particular sources. This finding is also relevant to research in using Large Language Models (LLMs) in healthcare^35^ wherein doctors may have to verify the information generated through LLMs and may not agree with some of the sources used by LLMs to generate content. Our findings imply that doctors’ perspectives should be considered when designing authoring tools for the development and sharing of medical content through AI tools like DTDs and LLMs.

The findings of the study also expand the knowledge of prior work in studying the adoption of AI-generated characters by people^2,6^. Prior work has explored how the use of AI-generated characters can support personalized well-being^2^ but has also highlighted mixed reactions of people to AI-generated characters, ranging from curiosity and amusement to fear and distrust^6^. Our findings expand this existing knowledge by providing nuanced details on potential benefits and concerns that might arise if we integrate AI-generated characters like DTDs into existing patient care. Due to shared identity, doctors believe that DTDs can make patients feel they are receiving specialized attention compared to current ways of sending non-personalized text messages. Patients will also be able to receive additional information that is usually not discussed due to lack of time during hospital visits like rare complications of medications and procedures. In addition, the digital nature of the DTDs allows patients to access the content at their own pace when convenient to them. However, as highlighted by doctors, while patients may benefit from DTDs, overuse of DTDs may reduce doctor–patient communication which is already a concern in existing patient care^18^. In addition, patients may lack knowledge of technology and may mistake DTDs for real doctors. To avoid such confusion and manage the expectations of patients, it is important for researchers to develop guidelines for introducing DTDs to patients. Additional research involving patients can help understand patient perceptions and the design of DTDs.

### Limitations and future work

Although this study makes a novel contribution to understanding the perceptions of doctors in utilizing DTDs in healthcare, limitations still exist. Firstly, the video shown in the interview was not a digital replica of a doctor. Doctors were asked to imagine if a similar video was created using their identity. In addition, the video did not fully demonstrate how users may interact with DTDs. Although the video provided sufficient details to allow the participants to understand the digital twin concept, there could still be gaps in watching a video of a random virtual character versus interacting with a DTD. Future research can investigate demonstrating the conversational capabilities of DTDs. In addition, in the video, participants were shown an example of a virtual character discussing lab tests to prime the participants to focus on DTDs usage in healthcare. However, this may have limited participants to only focus on utilizing DTDs in similar or related contexts. While the pilot studies helped us validate the clarity and relevance of the video in explaining the digital twins concept, the video was not validated with respect to how it may have influenced participants’ sentiments. Although the interview guide focused on both positive and negative sentiments which should have mitigated skewed sentiments about the technology during the analysis, a single video may have still influenced participants’ sentiments initially. Using the knowledge gained from the study, future research should highlight both positive usage and potential concerns of the technology in the study materials shown to participants. Finally, there is a potential for research biases. For example, the doctors involved in the research may provide more desirable responses, be more likely to participate in the experiment, and adopt new technologies in their practice. Future research should explore the perceptions of doctors who are late adopters of technologies.

### Conclusion

In conclusion, this study provides novel insights into doctors’ perceptions of utilizing DTDs in healthcare. It examines the use of doctors’ identities for DTDs, the benefits and concerns associated with DTD implementation, patient reception, financial considerations, and doctors’ willingness to adopt DTDs in their practice. The findings highlight the advantages of utilizing doctors’ identities for DTDs, including enhanced credibility and personalized care. However, concerns arise regarding the dissemination of misinformation using DTDs and disagreement with information sources used in developing DTD content. Doctors propose using random or alternative identities for DTDs, as AI-based characters can fulfill the required tasks without doctors’ identities. Doctors also anticipate that DTDs would benefit patients by providing a sense of specialized attention and delivering additional information. However, overreliance on DTDs may hinder doctor–patient communication, and patients’ limited technological knowledge may lead to confusion. Establishing guidelines for introducing DTDs to patients is essential. This study illuminates the multifaceted landscape of utilizing DTDs in healthcare, presenting both opportunities and challenges. The findings of this research are also relevant in the context of using LLMs and embodied conversational agents in healthcare. Incorporating doctors’ perspectives in the development and deployment of DTDs is pivotal for their responsible implementation.

### Supplementary Information

Supplementary Information.

Supplementary Video 1.

## Data Availability

The datasets generated during and/or analysed during the current study are available from the corresponding author on reasonable request.
