# Supplementary material for: Doctors’ perceptions of using their digital twins in patient care
Source: Sci Rep. 2023 Dec 7;13:21693. doi: 10.1038/s41598-023-48747-5 (PMC10709415; doi:10.1038/s41598-023-48747-5)
Supplement: Supplementary file 1 — Supplementary Information. [file 41598_2023_48747_MOESM1_ESM.docx]

Questions

1. Tell me a bit about yourself and your role as a physician
   1. How many patients do you see on average per day?
   2. How would you describe the workload?
      1. How does it affect the amount of time you spend with patients?
2. Can you pick a patient session from last week and walk me through what happened in one session?
3. How much time do you usually spend on one patient session?
4. Can you recall some of the instances when you thought you don’t have enough time to discuss all the information you wanted to discuss with patients?
   1. Can you describe the scenario?
      1. Could you describe me a session when you had limited time
   2. Do you mind sharing instances where you had to prioritize what to discuss with patients?
      1. How do you prioritize which information needs to be shared during the patient session?
      2. Usually what kind of information is not prioritized in the discussion?
         1. How is it delivered to the patient?
      3. Have you ever had a patient discuss they needed more time?

1. **Design probe**: Next I will show you an example of a video about the new technology we are exploring for patient care. It is called digital twins. Using this technology, we can use a digital human face to deliver information.
   1. This is a real human face but the behaviors are digitally generated by artificial intelligence. You can write anything in 65 different languages and use this face to verbalize them. They are called digital twins.
   2. Show a short video
      1. Would you like to see the video again?
      2. You can make it conversational
   3. Show the process of creation
   4. Have you heard/seen this kind of technology before today?
      1. How did you learn about them?
2. Now imagine this technology is used to create your digital twin - a digital representation of you. A digital twin of the doctor. So in that video, your face and voice will be used to create a digital twin. You can use that digital twin to augment your current practices and have conversations with your patient in different instances.
   1. What are your thoughts on that?
      1. In what instances would you use this technology in your practice?
      2. Can you describe some instances that you see would be advantageous?
      3. For example, if you were to use this for [lab testing] what would you have in this technology? How do you imagine this technology interacting with patients in your absence?
         1. roleplay
      4. If they mention earlier that they prioritize the information
         1. You mentioned time constraints stopping you from having detailed conversations with your patients. Can you think of some instances where this technology can be used to address that problem?
   2. What are some of the things that concern you?
      1. For any concerns raised
         1. Why does it concern you?
      2. If they raise concerns about what the agent will speak
         1. What if you could beforehand verify the responses provided by your digital twin?
      3. If legal implication concerns are raised
         1. Can you think of any scenarios where you could see legal concerns arise?
         2. Who do you think should be responsible?
      4. From patient’s side
3. How do you envision patients using your digital twin to communicate with patients?
4. Say if you were to introduce your digital twin to your patients, how would you describe it?
   1. Say a patient is not happy with what or how your digital twin described a few things and describes it to you during the patient visit. What are your thoughts on that?
      1. Relationship with patient
5. What constitutes a digital twin beyond facial and vocal resemblance?
6. If given a chance, would you be interested in creating a digital twin of yourself to communicate with your patients? If yes, why? If not, why not?
